# The mechanism on phosphorylation of Hsp20^Ser16^ inhibit GA stress and ER stress during OGD/R

**DOI:** 10.1371/journal.pone.0213410

**Published:** 2019-03-07

**Authors:** Tonglin Lu, Yongyi Zou, Xu Zhou, Wenna Peng, Zhiping Hu

**Affiliations:** 1 Department of Intensive Care Unit, Hunan Provincial People’s Hospital, Hunan Normal University, Changsha, Hunan, China; 2 Department of Neurology, Second Xiangya Hospital, Central South University, Changsha, Hunan, China; 3 Clinical Laboratory, Jiangxi Maternal and Child Health Hospital, Nanchang, Jiangxi, China; 4 Department of Rehabilitation, Second Xiangya Hospital, Central South University, Changsha, Hunan, China; University of Cincinnati College of Medicine, UNITED STATES

## Abstract

Recent research has demonstrated that small heat shock protein (sHsp) phosphorylation plays a variety of roles in neural cells. While the phosphorylation of serine 16 (Ser16) is blocked, Hsp20 no longer has neuroprotective effects. To further investigate the mechanism underlying this process, oxygen-glucose deprivation and reperfusion (OGD/R) was used with human SH-SY5Y cells and mouse N2a neuroblastoma cells. When SH-SY5Y and N2a cells were transfected with pEGFP-Hsp20(WT), pEGFP-Hsp20(S16A), and pEGFP-Hsp20(S16D) plasmids, the Golgi apparatus (GA) became more swollen and scattered, and many small fragments formed in the MOCK and S16A groups after OGD/R (P < 0.05). Meanwhile, the endoplasmic reticulum (ER) network was reduced, and the lamellar structure increased. However, these changes were not as obvious in the WT and S16D groups. Additionally, after OGD/R, Golgi Stress related protein contents were increased in the WT and S16D groups compared with the MOCK and S16A groups (P < 0.05). However, ER Stress related protein contents were decreased in the WT and S16D groups compared with the MOCK and S16A groups (P < 0.05). Our study demonstrates that Hsp20 phosphorylation on Ser16 protects against not only OGD/R-induced GA fragmentation in SH-SY5Y cells and N2a cells via Golgi stress but also OGD/R-induced ER structural changes in SH-SY5Y cells via ER stress. These findings suggest that Hsp20 is a potential drug target for ischemia stroke treatment.

## Introduction

Ischemic stroke is the most common type of stroke. Recent studies have shown that in China, the mortality rate of ischemic stroke patients is approximately 3.3–5.2% during the first month after disease onset and is 11.4–15.4% during the first year after disease onset. Early diagnosis, treatment, rehabilitation and prevention of ischemic stroke should be emphasized [1]. Currently, the ultra-early use of recombinant tissue plasminogen activator for intravenous thrombolysis is the most effective drug treatment to improve the outcome of acute ischemic stroke. However, thrombolytic therapy has certain time limitation and may induce many complications. Therefore, a better approach is needed to treat ischemic stroke. Reducing ischemic reperfusion injury is considered an effective measure for treating it.

In our previous studies, we found that small heat shock protein 20 (Hsp20) has protective effects in mouse N2a cells during ischemic reperfusion injury. After N2a cells were transfected with wild-type (WT) Hsp20 or serine 16 (Ser16) phosphorylation Hsp20 mutants (S16D) and treated with OGD/R, cell vitality was increased (P < 0.05), and the apoptosis rate was decreased (P < 0.01) in mutated cells compared with control cells. However, cell activity and the apoptosis rate were not significantly different in N2a cells transfected with the Ser16 non-phosphorylated Hsp20 mutant (S16A) compared with control cells [2].It is not that clear whether this phenomenon is related to Golgi Stress or ER Stress.

After ischemia reperfusion injury, as an important organelle, GA produces a series of responses to such blows, which is called "Golgi Stress". It has been confirmed that the morphology of GA changes significantly after cerebral infarction, its volume becomes larger, many small fragments and even particles form [3, 4]. Golgi stress related proteins includes Golgin160, Grasp65, Golgi microtubule-associated protein 210 (GMAP210) and so on. However, whether the protective effect of Hsp20 in the process of OGD/R is related to Golgi stress is not clear.

Proteins are synthesized and preliminarily processed by ER before the GA is further processed. Ischemia/hypoxia is known to induce ER stress [5] and increase mortality. Long time of protein mis-folding and oxidative stress after stroke will extend the time of ER stress. And these will lead to ER dysfunction and nerve cell death [6]. Conversely, if ER stress is inhibited, the damage caused by cerebral ischemia will be significantly reduced. So, the ER stress is thought to be a therapeutic target in ischemic/hypoxic injury. The signal pathways activated by ER stress are the following three, PKR-like ER kinase (PERK), Inositol-requiring enzyme 1(IRE1) and activating transcription factor 6 (ATF6) [7]. Its related factors include Glucose-regulated protein 78 (Grp78), C/EBP-homologous protein (CHOP), phos-Eukaryotic translation initiation factor 2A (eIF2alpha), and activation transcription factor 6 (ATF6), etc.

Recent research has demonstrated that Hsp20 overexpression significantly attenuates ischemia/reperfusion-induced apoptosis. When Hsp20 levels increase, OGD injury and N2a neuroblastoma cell reperfusion are prevented to a certain extent [2]. The GA Stress and ER Stress may participate in this process. Therefore, based on previous findings, the aims of this study were to determine the following: (1) whether OGD/R induces GA fragmentation and ER structural changes in SH-SY5Y cells, (2) whether Hsp20 phosphorylation on Ser16 protects the GA from OGD/R-induced fragmentation and the ER from structural changes in N2a and SH-SY5Y cells, (3) whether the factors associated with Golgi Stress play a specific role in the mechanism of GA fragmentation, and (4) whether the factors associated with ER stress play a certain role in the mechanism of ER structural changes. In order to be closer to human physiology and pathology, mouse N2a and human SH-SY5Y cell are all used in this research. We found GA fragmentation and ER structural changes in human SH-SY5Y cells after OGD. Hsp20 protected against OGD/R-induced GA fragmentation and ER structural changes in N2a and SH-SY5Y cells. When the GA was more markedly fragmented, Golgi stress related protein levels were more substantially decreased. The protective role of Hsp20 in decreasing ER structural changes is thought to be mediated though the ER stress pathway.

## Materials and methods

### Mouse N2a neuroblastoma and human SH-SY5Y cell culture

Human SH-SY5Y cells and mouse N2a cells were purchased from the National Key Laboratory of Medical Genetics, Central South University. N2a and SH-SY5Y cells were revived, cultured, digested, passaged and cryopreserved by conventional methods. Dulbecco’s modified Eagle’s medium (DMEM) was used with 10% fetal bovine serum (Gibco BRL) added. Cells were cultured in a cell incubator containing 5% CO_2_ at 37°C, and the culture medium was changed every three days.

In this experiment, N2a cells were first selected for the pre-experiment, and it was found that Hsp20 could inhibit Golgi stress. Then, human SH-SY5Y cells, which were closer to human physiology and pathology, were selected for the experiment. But based on the logic of the whole article, the results of N2a cells and SH-SY5Y cells were integrated into each paragraph.

### Plasmid construction and transfection

Total RNA was isolated from N2a cell cultures using TRIzol (Invitrogen). A reverse transcription kit (Promega) was used to perform reverse transcription. Primers with restriction nuclease sites for BamHI and EcoRI in the 5'-terminal region contributed to Hsp20 acquisition. The following primer sets were used and are the same as those described in our previous research [8]: Hsp20-WT forward, 5’-GCGAATTCATGGAGATCCCCGT GCCTGTGCA-3’; Hsp20-S16A forward 5’-GCGAATTCATGGAGATCCCCGTGCCTGTGCAGCCTTCTTGGCTG CGCCGTGCTGCAGCTCCTTTA-3’; Hsp20-S16D forward 5’-GCGAATTCATGGAGATCCCCGTGCCTGTGCAGCC TTCTTGGCTGCGCCGTGCTGACGCTCCTTTA-3’; and Hsp20 reverse 5’-GCGGATCCGCCTTGGCAGCAGGTGG TGACGGA-3’. PCR products were cloned into pEGFPN1 vectors using EcoRI and BamHI. After being identified by sequencing, the pEGFPN1 vector plasmids and recombinant plasmids were transfected into N2a and SH-SY5Y cells. Cells transfected with pEGFPN1 vector plasmids represent the MOCK groups.

Cell transfection was performed according to the instructions of Lipofectamine 2000 (the amount of plasmids and liposomes added in the following sections was the amount of 24 well plates per well). Firstly, 50 μL Opti-MEMI (serum-free medium) was added to a 1.5ml centrifuge tube, and then 0.8 g plasmid was added to it. The liquid in the centrifuge tube was homogenized by hand and incubated at room temperature for 5 minutes. Secondly, 50 μL -MEMI (serum-free medium) was added to another 1.5ml centrifuge tube, and 1.6 μL liposome was added. The liquid in the centrifuge tube was homogenized by hand and incubated at room temperature for 5 minutes. Thirdly, mix and homogenize the two solutions, and incubate at room temperature for 20 minutes. Fourthly, during the mixed incubation of the two solutions, the medium in the 24-well plate was absorbed and washed with 1ml DMEM medium (without serum) once, and then 0.5ml DMEM (without serum) was added to each well. At last, the plasmid /Lipofectamine 2000 liposome solution was incubated for 20 minutes and gently added into 12 Wells. Culture for four to five hours in an incubator at 37 degrees Celsius and 5 percent carbon dioxide, followed by 24 hours in a complete medium. The above methods were used to transfect peGFP-N1, Hsp20-WT, and each mutant into SH-SY5Y cells. Hsp20 expression in the transfected cells was assayed by immunofluorescence and westernblotting.

### Oxygen-glucose deprivation and reperfusion (OGD/R)

Briefly, phosphate-buffered saline (PBS) was used to wash SH-SY5Y cells (differentiated) and N2a cells twice, and the medium was replaced with deoxygenated glucose-free Hanks' balanced salt solution (Invitrogen) [9]. The cells were then placed in a hypoxia chamber (Forma Scientific), which was filled with a gas mixture (95% N_2_, 5% CO_2_), and incubated at 37°C. After 4 hours, the medium was replaced with DMEM/F-12 containing 10% FBS, and the cells were cultured under normoxic culture conditions for 0, 6, 24, and 36 hours.

### Measurement of apoptosis

To evaluate the possible cellular effects induced by OGD/R, annexin V and PI staining was used to assess apoptosis in SH-SY5Y cells. A single sample represents a collection of data in a group. This was repeated for three times, and the average was taked for statistics. First, SH-SY5Y cells were transfected with pEGFP or pEGFP-Hsp20 plasmids for 36 hours. Next, after OGD for 4 hours, the cells were reperfused for 0, 6, 24 or 36 hours. An annexin V-FITC/PI apoptosis detection kit (MB-CHEM, no. M3021) was used to detect apoptosis. Briefly, SH-SY5Y cells were collected and washed once with PBS (4°C). Annexin V labeling solution was prepared for each specimen (10^5^−10^6^ cells), which included 10 μl of 10 × binding buffer, 5 μl of annexin V-FITC, and 85 μl of distilled water. After the cells were suspended in labeling solution and incubated in the dark for 15 minutes at room temperature, 10 μl of PI was added. Finally, the incubation solution was diluted with 400 μl of 1 × binding buffer. The percentage of apoptotic cells was detected by flow cytometry (BD FACS Aria), while the flow cytometry analysis was evaluated by FlowJo software.

### MTT assay

The MTT assay was performed to detect cell viability following treatment with OGD/R. Each group of SH-SY5Y cells were seeded onto ninety-six-well plates, with 100 μl (approximately 1 x 10^4^ cells) in every well. To each well, 50 μl of 1× 3-(4,5-dimethylthiazol-2-yl)-2,5-diphenyl tetrazolium bromide (MTT, cat: KGA312 1000 assays, Beijing Sciencbio Biotechnology Co., Ltd., China) was added into each well. Then, the cells were incubated in an incubator for 4 hours at 37°C to revert MTT into insoluble formazan. The supernatant was aspirated, and 150 μl of dimethylsulfoxide (DMSO, Sigma-Aldrich Inc, St. Louis, MO, USA) was added to each well to dissolve the formazan. The optical density of each well was measured at 570 nm by a microplate reader (BioRad, Hercules, CA, USA). The OD value minus the control OD value of each test well (complete medium with MTT, no cells) was used as the test well OD value. Normal cells were used for the control group. Cell viability was calculated as follows: OD value of test well/OD value of control group × 100%.

### Immunofluorescence staining

Mouse N2a neuroblastoma cells and human SH-SY5Y cells were transfected with pEGFP or pEGFP-Hsp20 plasmids for 36 hours. After 36 hours of transfection, the cells were exposed to OGD for 4 hours and then reperfused for 0, 6, 24 or 36 hours. Paraformaldehyde was used to fix the cells for 30 minutes, and PBS (pH 7.4) was used to wash them 3 times. The cells were incubated with TGN38 (Santa Cruz, no. 33784), GM130 (BD, no. 610823) and Calnexin (Sigma, no. 4631) primary antibodies overnight at 4°C. The next day, fluorescein-conjugated anti-rabbit IgG (1:400; National Key Laboratory of Medical Genetics, Central South University) were incubated with the cells for 1 hour. To observe nuclear morphology, N2a and SH-SY5Y cells were stained with 1 μg/mL 4’, 6-diamidino-2-phenylindole (DAPI) (Vector Laboratories, Burlingame, CA, USA). Slides were washed with deionized water, mounted with glycerol, and examined with an Olympus confocal fluorescence microscope. Fluorescence images were obtained with identical exposure settings.

### Western blot analysis

Human SH-SY5Y cells were also transfected with pEGFP or pEGFP-Hsp20 plasmids for 36 hours. After 36 hours of transfection, the cells were exposed to OGD for 4 hours and then reperfused for 0, 6, 24 or 36 hours. Total protein was isolated from SH-SY5Y cells using 2× SDS sample buffer (2% SDS, 10% glycerol, and 63 mM Tris-HCL). The protein concentration was detected using Pierce BCA protein assays (Thermo Scientific, #23225). Samples (20–40 μg of protein) were transferred to SDS/PAGE by electrophoresis and then to PVDF membranes. The membranes were blocked in TBST buffer containing 1× PBS, 0.1% Triton X-100 and 5% nonfat milk at room temperature for 1 hour. Then, the blots were incubated with anti-GMAP210 (SC-135928, 1:200, SANTA), Grasp65 (SC-19481, 1:500, SANTA), Golgin160 (21193-1-AP, 1:1000, Proteintech), Grp78 (ab108613, 1:200, Abcam), CHOP (ab11419, 1:2000, Abcam), phos-Eif2α1 (3398, 1:1000, CST), ATF6 (BS-1634R, 1:500, BIOSS) and GAPDH (SC-365062, 1:800, SANTA) primary antibodies at room temperature for 1–2 hours. The PVDF membrane was washed 2–3 times with PBST. Then, PBST was added to the bleaching table for 15 minutes and discarded. The membrane was then washed thrice. The membrane was incubated with secondary antibody (1:4000; goat anti-mouse IgG/HRP, rabbit anti-goat IgG/HRP, or goat anti-rabbit IgG/HRP; Abcam) at room temperature for 1 hour and then washed with TBST 3 times for 10 minutes intervals, followed by 2 washes with TBS for 10 minutes intervals.

The PVDF membrane was fully immersed in the chemiluminescence ECL reagent for 3–5 minutes. In the darkroom, the X-ray film was laid on the PVDF film, close to the front, and the developing clip was buckled. Different exposure times were selected according to the intensity of the fluorescence signal. X-rays were removed after exposure.

### Measurement of GA volume

Image J software was used to test GA volume. After opening the image to be processed, the pixel size of the bar is first calculated. Click the line tool, then draw a straight line in the picture according to the length of bar. After drawing the line, click "Measure" in the drop-down menu of "Analyze". The result box appears. Next, set the scale. Click "Set Scale" in the drop-down menu of "Analyze". Fill in the table that pops up. "Distance in pixels" was calculated by the software in the preceding step. "Known distance" is the length of the line segment measured in the previous step (that is the length of bar). The default value of "Pixel aspect ratio" is 1. The specific ratio can be viewed through "image J-> Properties". Thirdly, start measurement after setting up. Click the heart on the software interface, and then circle the boundary of GA on the image. After the boundary is drawn, "Measure" under "analyze" is selected again. The calculated area can be seen in "result".

### Quantitative and statistical analysis

Data are expressed as the means ± SD, and P < 0.05 was considered statistically significant. Selected bands in western blotting were analyzed by Gel pro4.0 optical density analysis software, and integrated optical density (IOD) values were measured. Quantitative data are based on at least 3 separate trials with triplicate samples.

For the two groups, two-tailed Student’s t-tests were used. For Comparison between 3 groups or more, the pairwise Comparison within the group was performed using one-way ANOVA or Tukey's test. The Comparison between treatment groups and the control group were performed using Dunnett's Multiple Comparison test.

## Results

### Detection of cell apoptosis rate and viability

**Fig 1:**
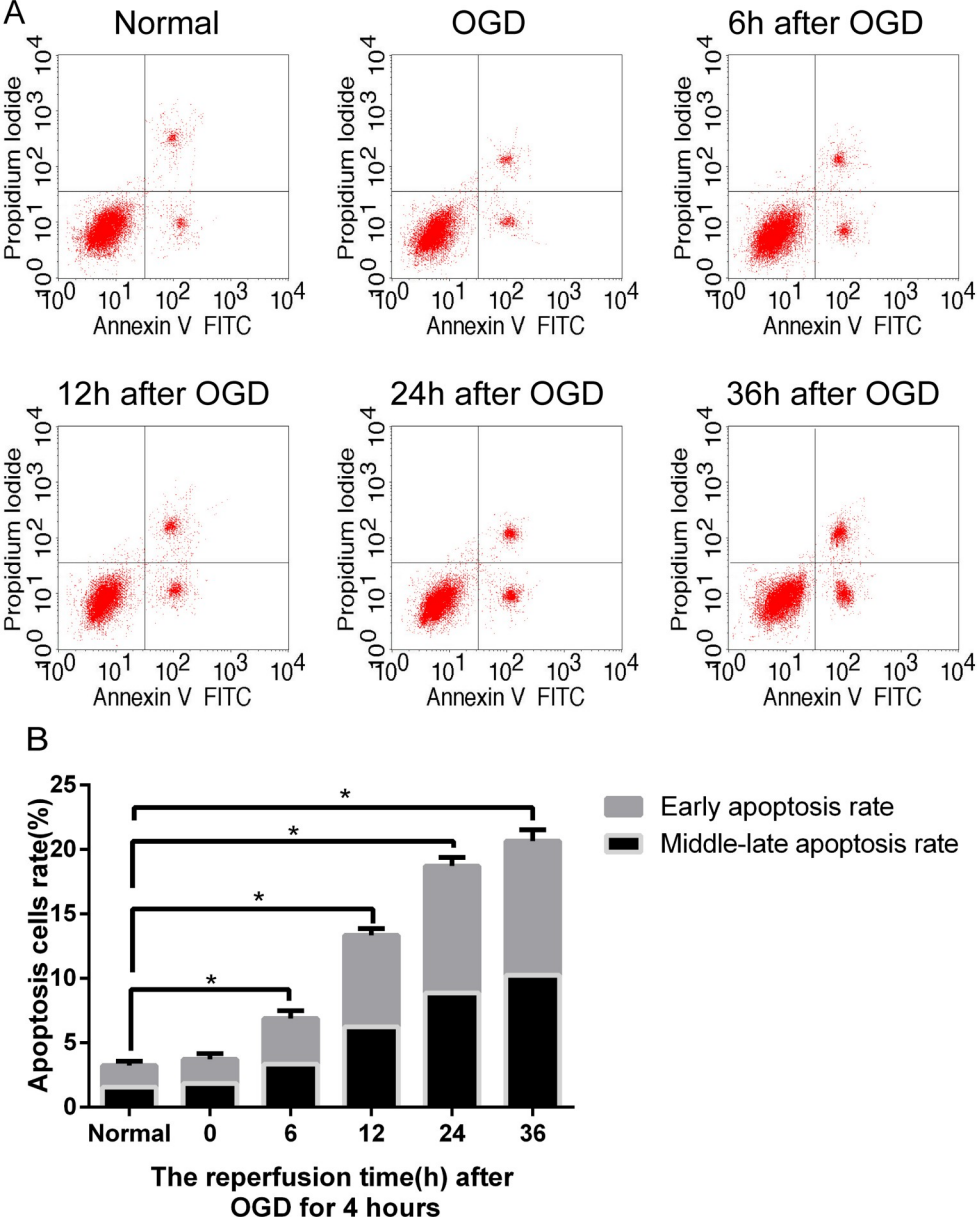
Apoptosis in non-transfected human SH-SY5Y cells after OGD/R. (A) In the figures, the upper right represents the middle and late stages of apoptosis, the lower right represents the early apoptotic rate, and the two combined equal the total apoptosis rate. (B) No significant difference was found between the normal and 0-hour reperfusion cells following 4 hours of OGD. However, after 6, 12, 24, and 36 hours of reperfusion, the proportion of apoptotic cells was significantly increased compared with that under normal conditions. Data are presented as the means ± SD (n = 3); * indicates P < 0.05.

**Fig 2:**
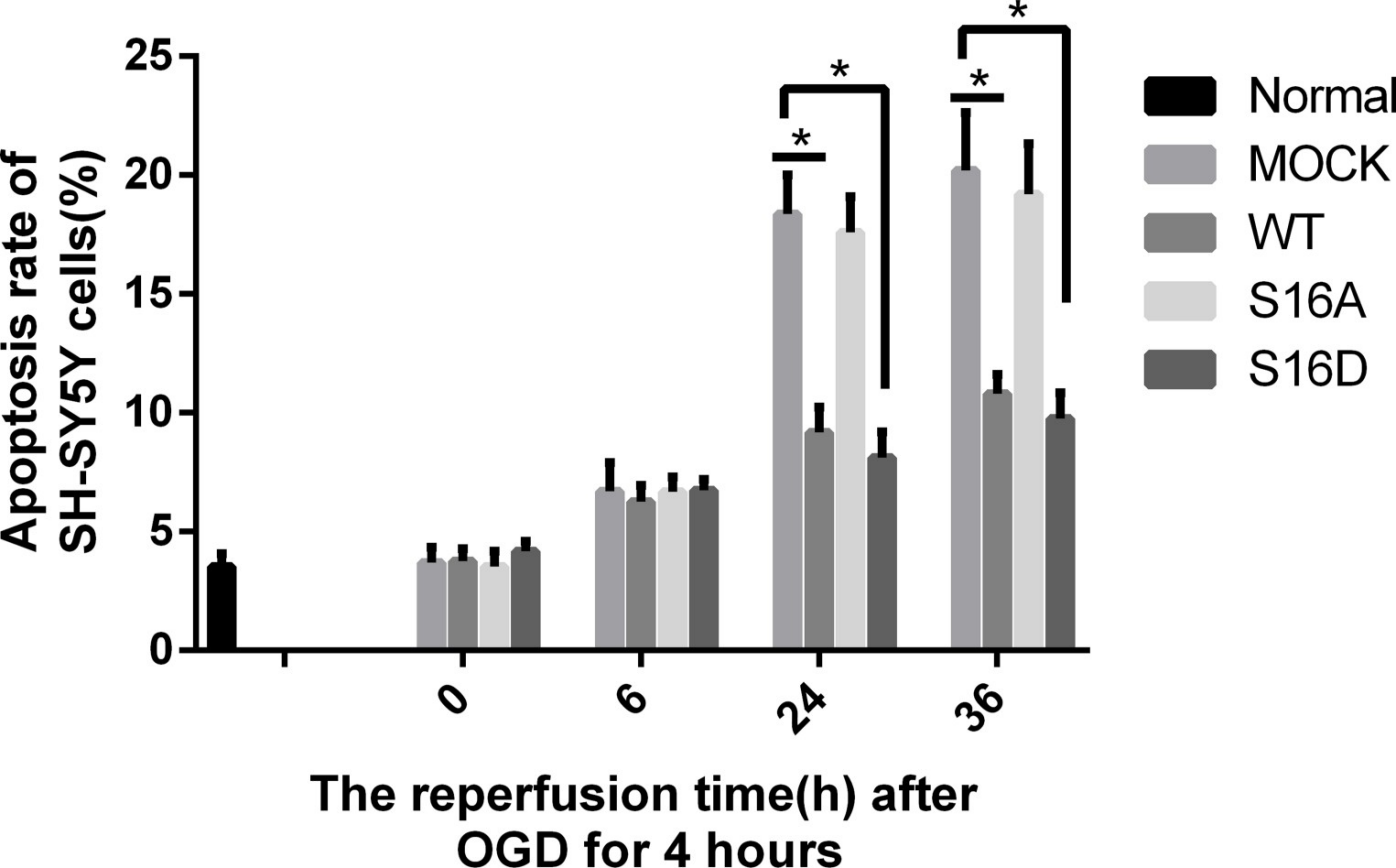
The apoptosis rate of human SH-SY5Y cells transfected with pEGFP-N1, WT, S16A and S16D plasmids after OGD/R. Data are presented as the means ± SD (n = 3); * indicates P < 0.05.

Flow cytometry was used to detect the apoptosis rate of SH-SY5Y cells. The apoptosis rate of normal cells was 3.24%. After OGD, the apoptosis rate of SH-SY5Y cells was 3.71%. After reperfusion for 6, 12, 24 or 36 hours following OGD, the apoptosis rate of SH-SY5Y cells was increased to 6.88%, 13.35%, 18.71% and 20.65%. SH-SY5Y cell apoptosis increased with perfusion time after 4 hours of OGD (Fig 1) (P < 0.05). After reperfusion for 24 or 36 hours, the apoptosis rates of SH-SY5Y cells were decreased in the WT and S16D groups compared with the MOCK groups (P < 0.05). In addition, there was no significant difference between the MOCK and S16A groups (Fig 2).

**Fig 3:**
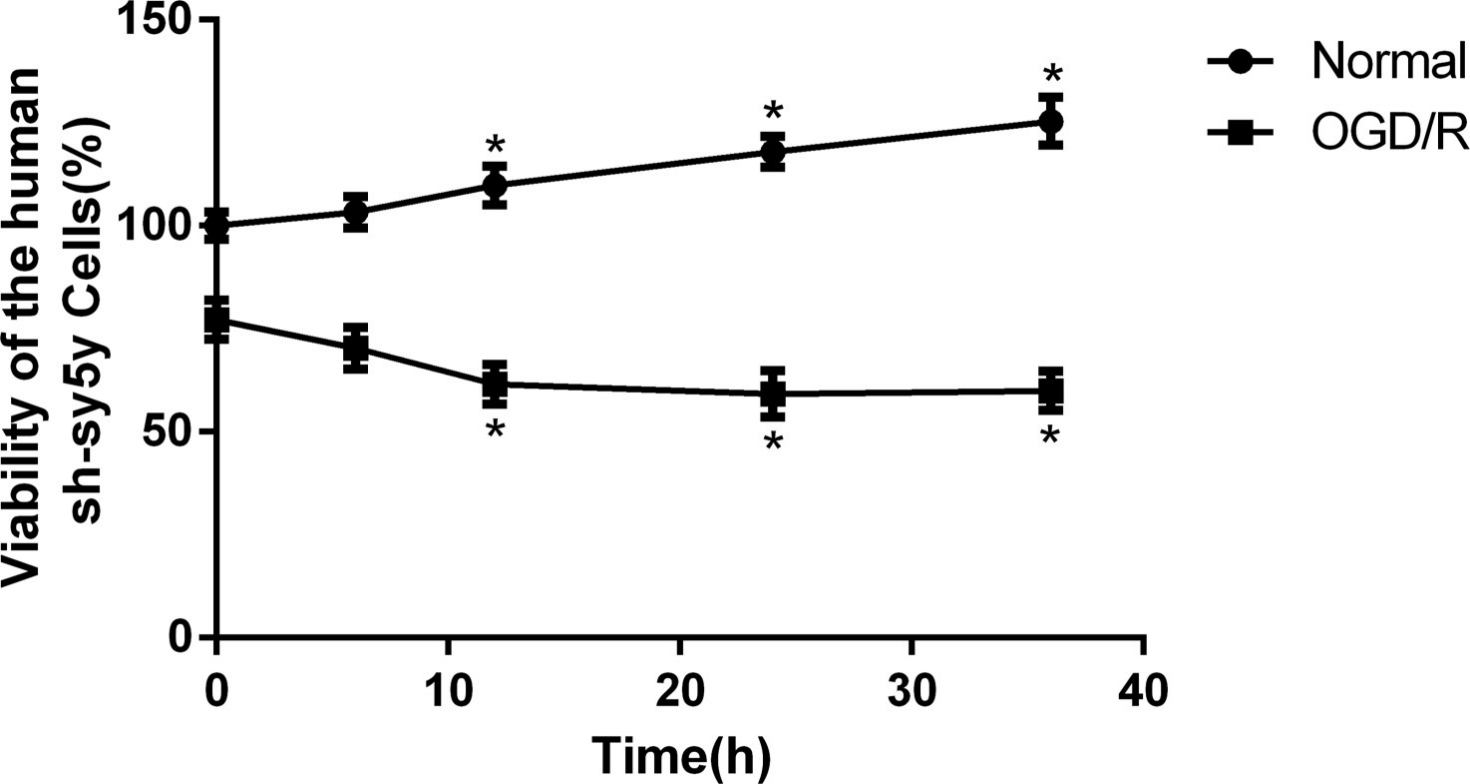
The viability of human SH-SY5Y cells. After 4 hours of OGD, viability was decreased with reperfusion time in the OGD/R groups compared with the normal groups (P < 0.05). Data are presented as the means ± SD (n = 3); * indicates P < 0.05.

**Fig 4:**
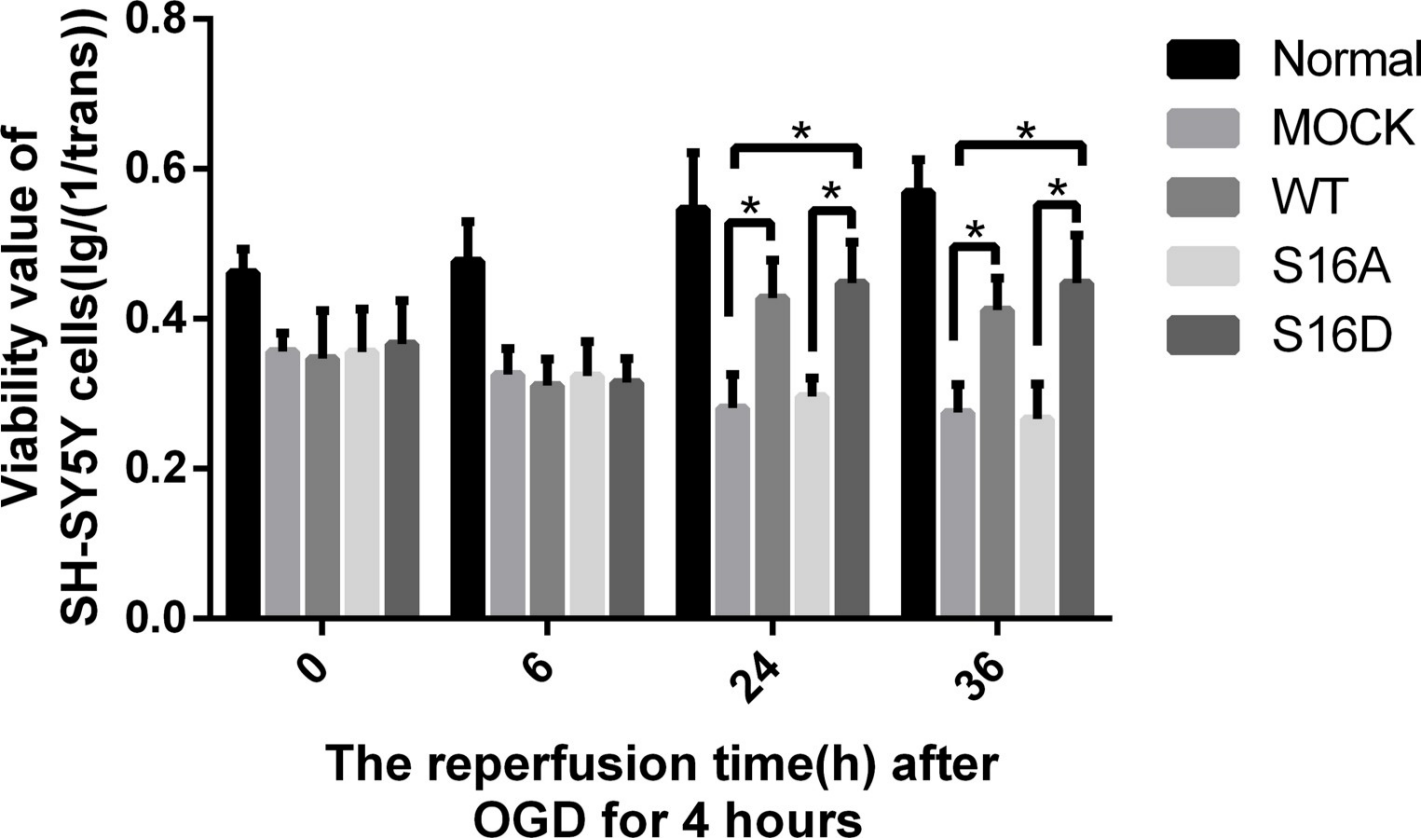
The OD value (MTT) after OGD/R of human SH-SY5Y cells transfected with pEGFP-N1, WT, S16A and S16D plasmids. Data are presented as the means ± SD (n = 3); * indicates P < 0.05.

SH-SY5Y cell viability was estimated by MTT assays. The viability of normal SH-SY5Y cells at 6, 12, 24 and 36 hours was 103.25%, 109.76%, 117.97%, and 105.37%. However, after reperfusion for 0, 6, 12, 24 or 36 hour following OGD for 4 hours, SH-SY5Y cell viability was 77.22%, 70.27%, 61.54%, 59.17% and 59.99%. The viability of SH-SY5Y cells in OGD/R groups decreased with the perfusion time after 4-hour of OGD compare to the Normal groups (Fig 3) (P < 0.05). After reperfusion for 24 hours and 36 hours, the cell viability of SH-SY5Y cells increased in WT and S16D groups compare to MOCK groups (P < 0.05). In addition, there is no significant difference from MOCK groups and S16A groups (Fig 4).

### Hsp20 protects against GA fragmentation in mouse N2a and human SH-SY5Y cells

**Fig 5:**
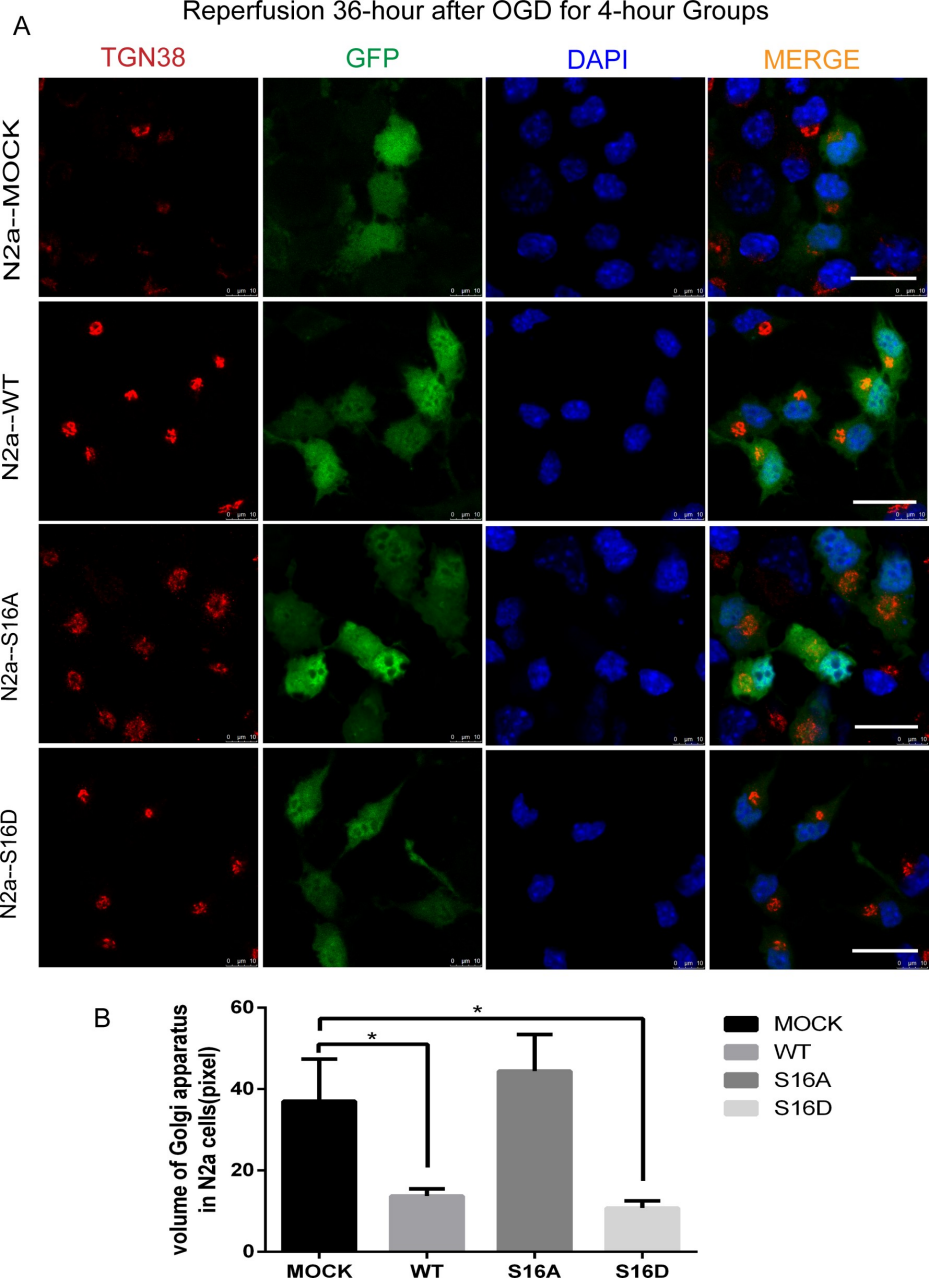
The morphological changes in the Golgi apparatus of N2a cells after reperfusion for 36 hours following 4 hours of OGD in each group. Ten cells in three images for each group were used for statistical analysis. (A) Trans-Golgi network protein 38 (TGN38) is a specific protein marker for the Golgi apparatus. Red represents TGN38, green represents fluorescently labeled plasmids, and blue represents DAPI-labeled nuclei. After OGD/R, the Golgi apparatus became more swollen and fractured into many small fragments. This morphological change was more pronounced in S16A and MOCK groups compared with WT and S16D groups. The scale bar = 20 μm. (B) The GA volume in each group. * indicates P < 0.05.

**Fig 6:**
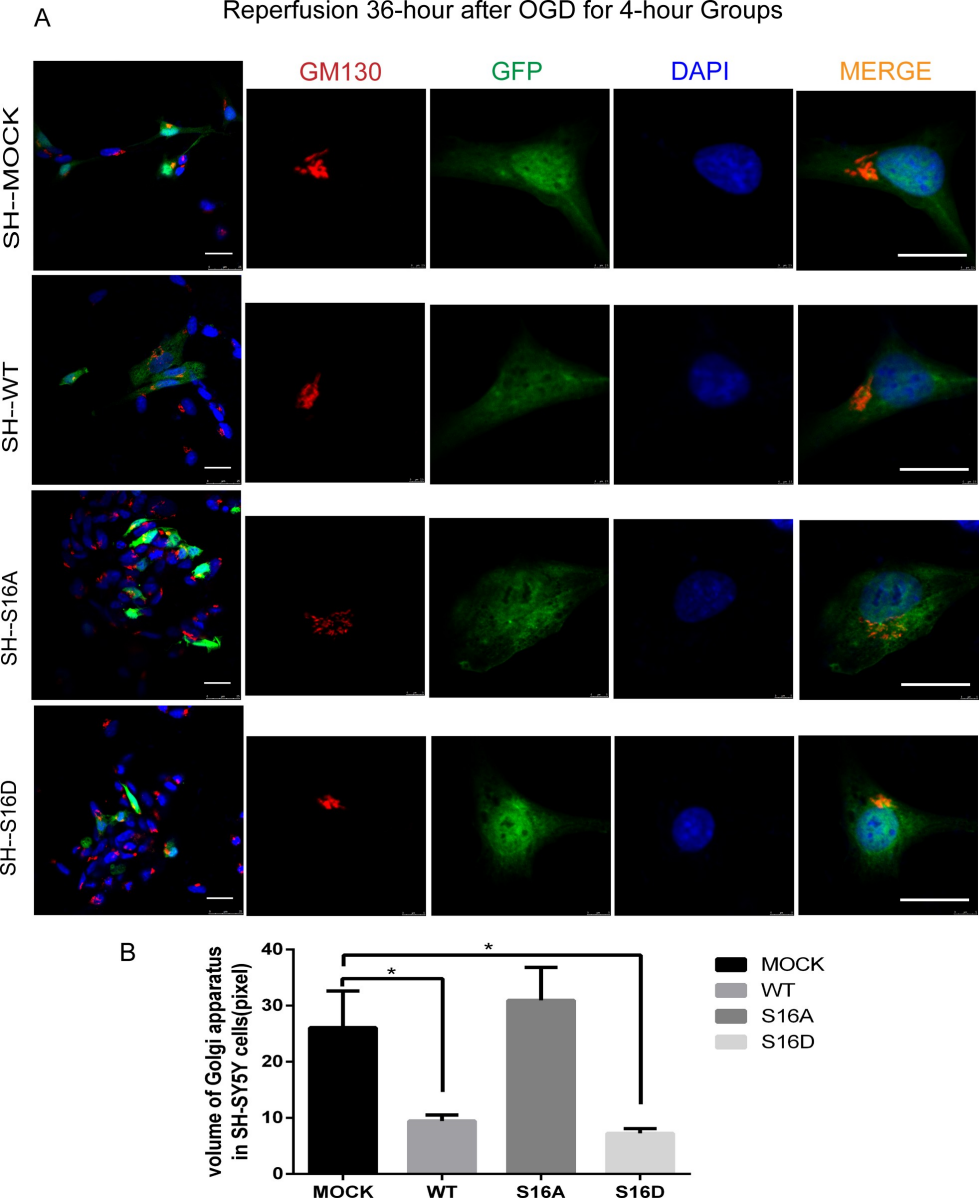
The morphological changes of the Golgi apparatus in SH-SY5Y cells after reperfusion for 36 hours following OGD for 4 hours in each group. Ten cells in three images for each group were used for statistical analysis. (A) GM130, a stromal protein of the cis-Golgi apparatus, participates in maintaining Golgi morphology. Red represents GM130, green represents fluorescently labeled plasmids, and blue represents DAPI-labeled nuclei. After OGD/R, the Golgi apparatus fractured into many small fragments. The degree of Golgi apparatus fragmentation was reduced in the WT and S16D groups compared with the MOCK and S16A groups of human SH-SY5Y cells. The scale bar = 20 μm. (B) The GA volume in each group. * indicates P < 0.05.

To understand the neuro-protective role of non-phosphorylated and phosphorylated Hsp20 in OGD treatment-induced apoptosis, we examined the respective effects of a phospho-mimetic Hsp20 mutant (S16D) and non-phosphorylated Hsp20 (S16A) on mouse N2a and human SH-SY5Y cells. N2a and SH-SY5Y cells were transfected with pEGFP-Hsp20 (S16D) or pEGFP-Hsp20 (S16A) for 36 hours and then re-perfused for 24 or 36 hours following 4 hours of OGD. After OGD/R, GA morphology changed and the whole structure was cleaved, forming many small, fragmented structures. However, compared with pEGFP-N1 transfection, pEGFP-Hsp20 (S16D) transfection greatly decreased the amount of GA fragmentation. GA volume was smaller in the WT and S16D groups than in the MOCK groups (P < 0.05). In contrast, compared with control cells, pEGFP-Hsp20 (S16A)-transfected cells did not presented with any significant differences in OGD-induced GA fragmentation (pEGFP-N1) (Figs 5 and 6) (P < 0.05).

### Effects of Hsp20 on golgi stress related proteins

**Fig 7:**
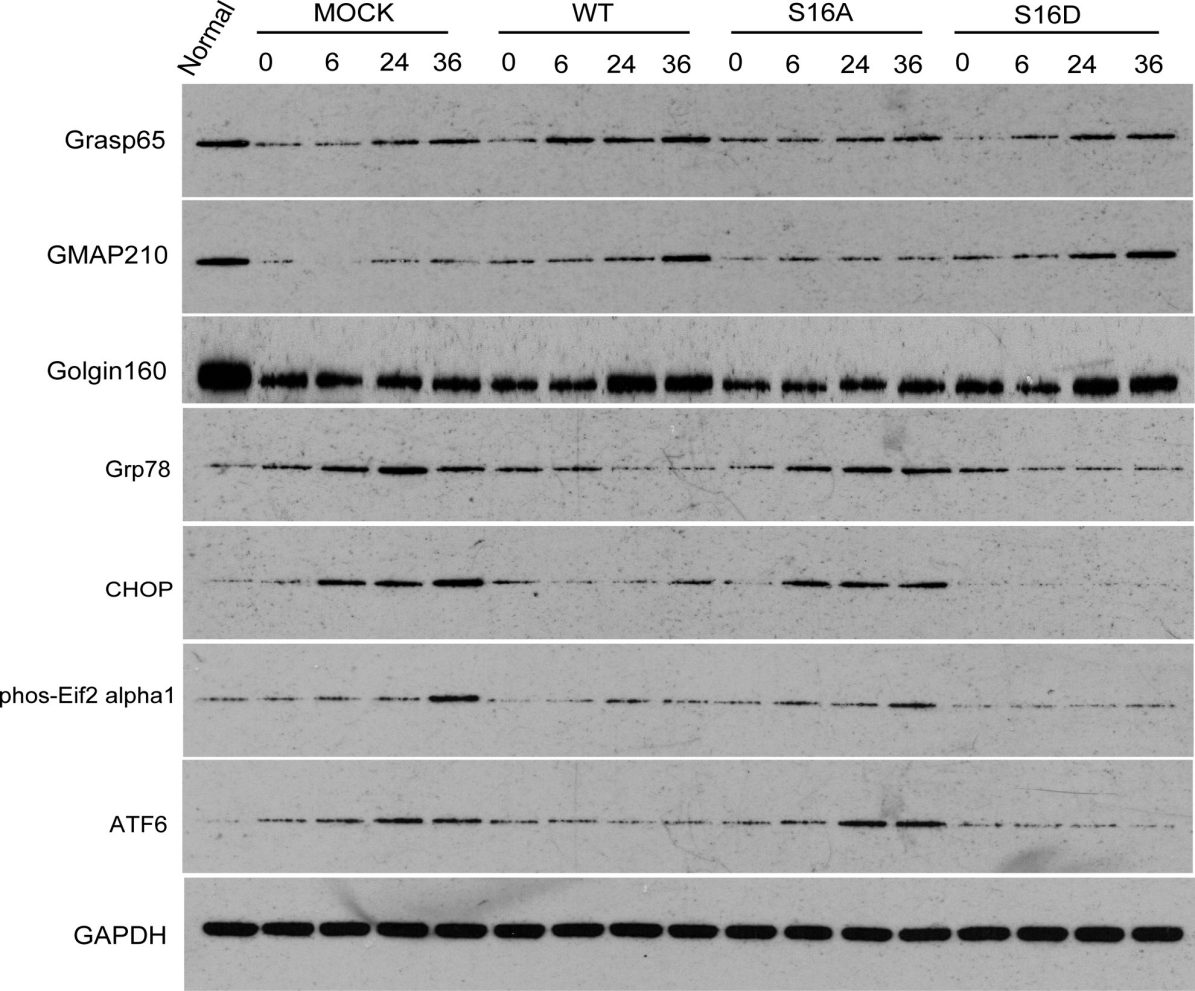
Grasp65, GMAP210, Golgin160, Grp78, CHOP, phos-Eif2α1 and ATF6 protein content after OGD/R in each group. GAPDH is the internal reference.

**Fig 8:**
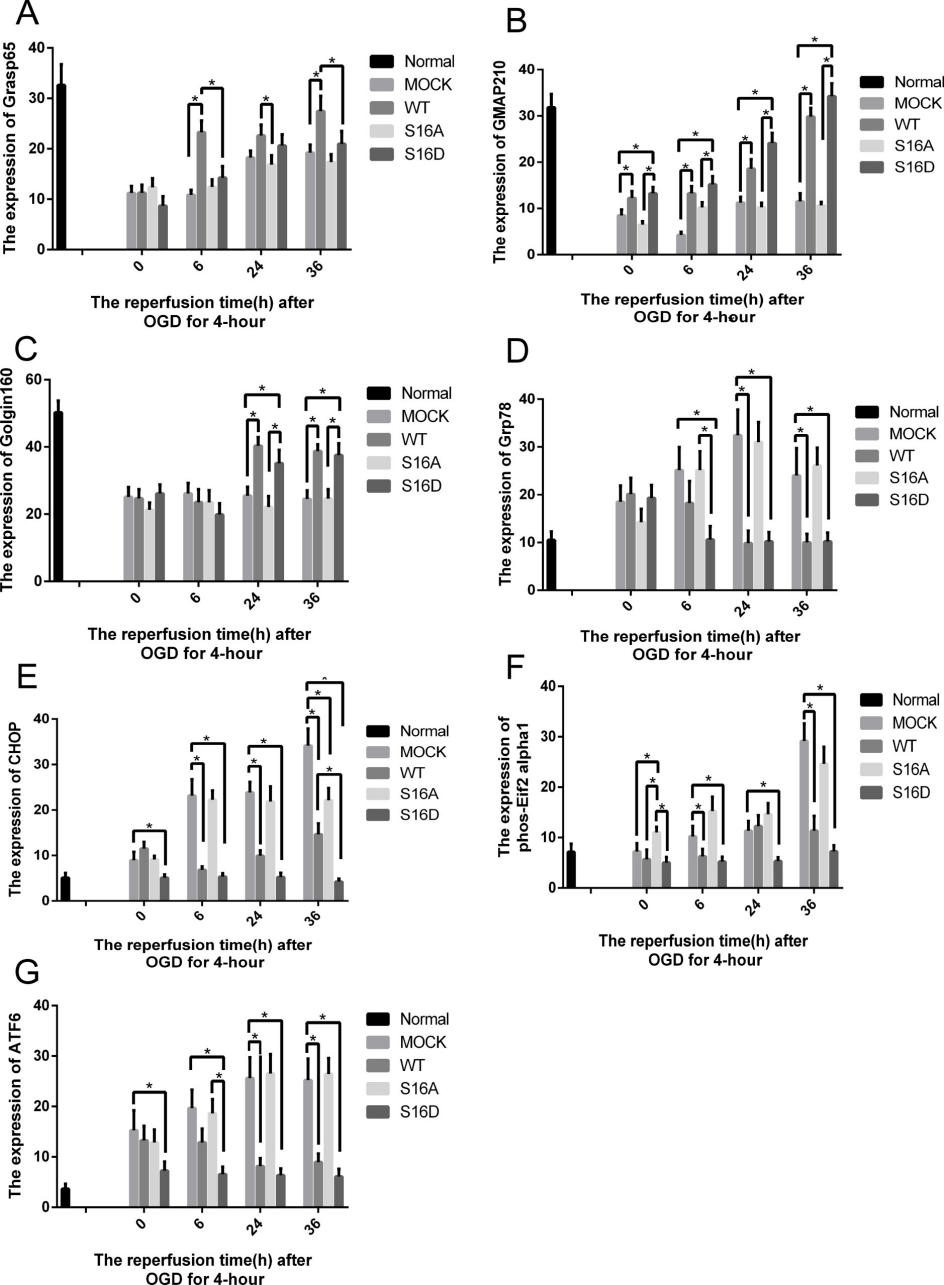
The change in Grasp65, GMAP210, Golgin160, Grp78, CHOP, phos-Eif2α1 and ATF6 expression after OGD/R in each group. In the 24- and 36-hour groups, GMAP210 and Golgin160 protein content in the MOCK groups was significantly different from that in the WT and S16D groups. In the 6-hour groups, Grasp65 and GMAP210 protein content in MOCK groups was significantly different from that in WT groups. The asterisks signify that the protein content in the WT and S16D groups was different from that in the MOCK groups. * indicates P < 0.05.

To determine how Hsp20 reduced OGD-induced GA fragmentation, we examined Grasp65, GMAP210, and Golgin160’s protein contents. These are all Golgi Stress related proteins. Human SH-SY5Y cells were transfected with pEGFP-Hsp20 (WT), pEGFP-Hsp20 (S16D), pEGFP-Hsp20 (S16A), or pEGFP plasmid for 36 hours and then treated with 0-, 6-, 24-, or 36-hour reperfusions following 4 hours of OGD. Grasp65, GMAP210, and Golgin160 expression was significantly decreased in the OGD-treated MOCK and S16A groups compared with the normal group (P < 0.05). GMAP210 expression was gradually increased with reperfusion time in the WT and S16D groups. Compared with pEGFP-N1 transfection, transfection with pEGFP-Hsp20 (S16D) greatly increased GMAP210 and Golgin160 protein content (P < 0.05). In contrast, Grasp65, GMAP210, and Golgin160 expression was similarly in pEGFP-Hsp20 (S16A)-transfected cells compared with control cells (pEGFP-N1-transfected cells) (Figs 7 and 8).

### Hsp20 inhibits OGD/R-induced ER structural changes

The four plasmids, including pEGFP-N1 (MOCK), WT, S16A and S16D, were transfected into human SH-SY5Y cells. After 36 hours, the cells were fixed by paraformaldehyde. To observe ER structure, Calnexin was stained using immunofluorescence methods. The ER is a closed network in the endometrium and has a high degree of polymorphisms.

**Fig 9:**
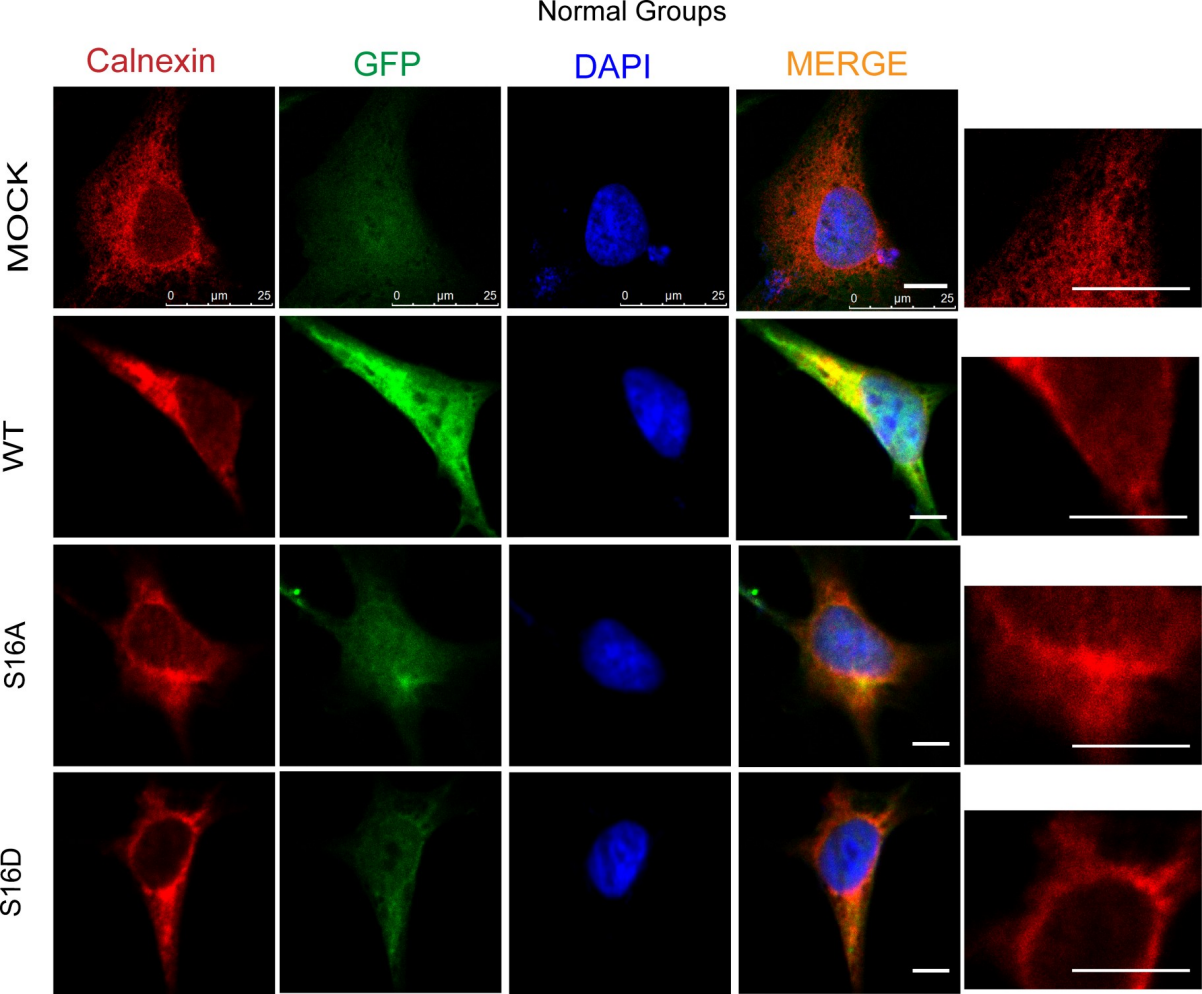
Endoplasmic reticulum morphology in SH-SY5Y cells transfected with pEGFP-N1, WT, S16A and S16D plasmids. Red represents Calnexin, green represents fluorescently labeled plasmids, and blue represents DAPI-labeled nuclei; the far right is a magnified display of the endoplasmic reticulum. The scale bar = 10 μm.

**Fig 10:**
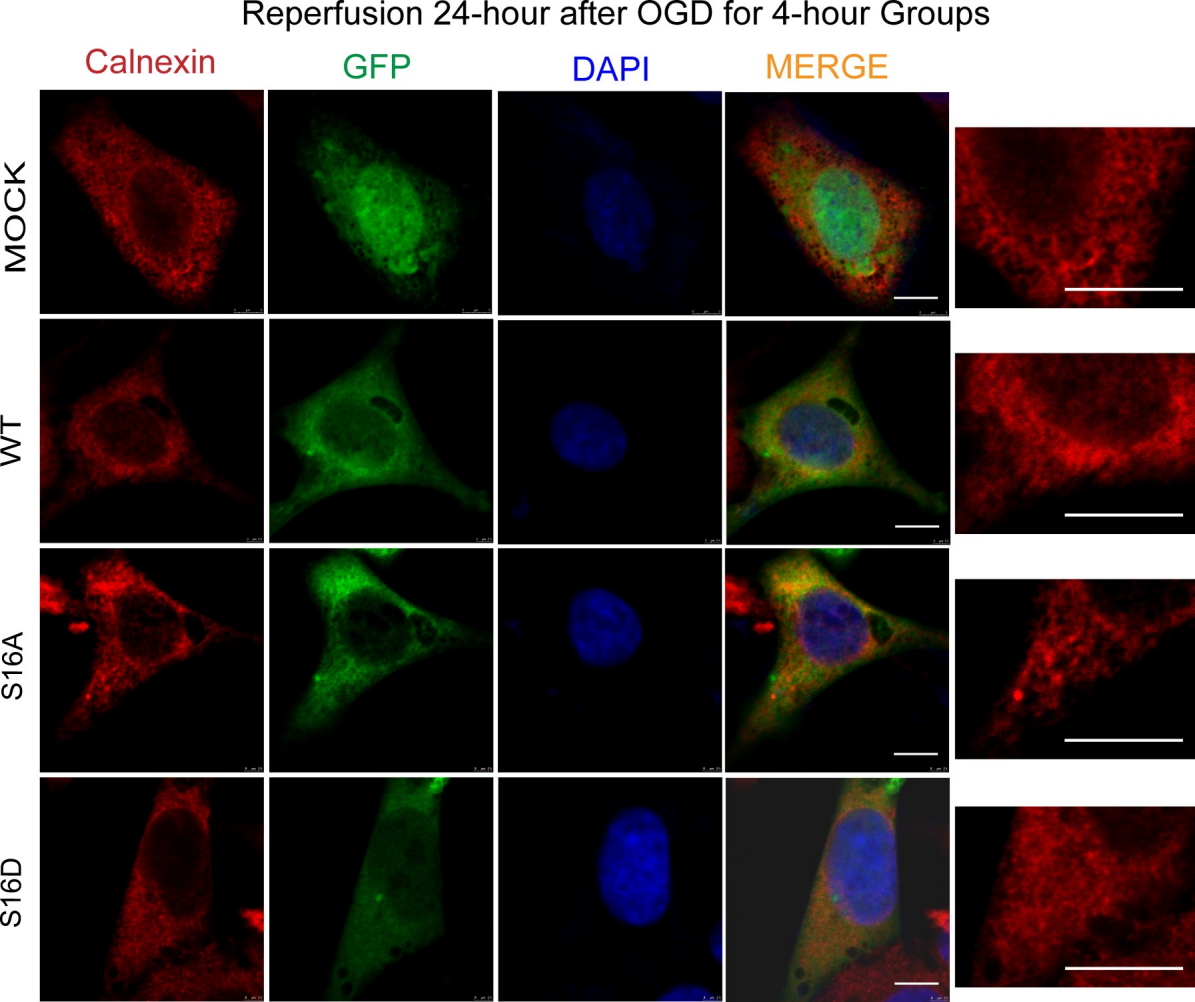
The endoplasmic reticulum structural changes in each group after a 24-hour reperfusion following OGD for 4 hours. The distribution of Calnexin in human SH-SY5Y cells is shown. Red represents Calnexin, green represents fluorescently labeled plasmids, and blue represents DAPI-labeled nuclei; the far right is a magnified display of the endoplasmic reticulum. The scale bars = 10 μm.

The rough ER (RER) is a flat sac, arranged in a neat, membrane-enclosed space called the ER cavity (lumen). ER structure was not different among the four groups (Fig 9). However, after SH-SY5Y cells were re-perfused for 24 hours following 4 hours of OGD, ER structure changed significantly. The ER network reduced and expanded, and cisternae became thicker. In the WT and S16D groups, after SH-SY5Y cells experienced OGD/R treatment, ER morphology changed. The grid pattern was reduced, and the laminar shape was increased. However, ER morphological changes were smaller in the WT and S16D groups than in the MOCK and S16A groups (Fig 10). Thus, the morphological structure of the ER was better stabilized.

### ER stress-related factors

Human SH-SY5Y cells were transfected with pEGFP-N1 vector (MOCK), pEGFP-Hsp20 (WT), pEGFP-Hsp20 (S16A) and pEGFP-Hsp20 (S16D) for 36 hours and then re-perfused for with 0, 6, 24 and 36 hours following 4 hours of OGD. Grp78, CHOP, phos-Eif2α1 and ATF6 expression was detected at these time points. These factors are all proteins associated with ER stress pathways. All these factors were significantly increased in the MOCK groups compared with the normal cell group. After reperfusion for 6, 24, or 36 hours following OGD for 4 hours, Grp78, CHOP, phos-Eif2α1 and ATF6 expression was higher in the MOCK and S16A groups than in the WT and S16D groups (P < 0.05, Figs 7 and 8).

## Discussion

Hsp20 is a stress-related protein that is widely expressed in the cerebral cortex. After hypoxia, the protein content of Hsp20 in hippocampal tissues increased rapidly, which was one of the initial landmark events of hypoxia. Previous studies have confirmed that Hsp20 has protective effects on nerve cells during oxy-glucose deprivation/reperfusion, but it is not clear whether the neuro-protective effects of Hsp20 are related to GA. Golgi stress represents GA's ability to respond to homeostasis in cells. However, if the homeostasis in cells exceeds its ability to respond, Golgi fragmentation, related gene and protein changes will occur [3, 4]. In this study, we found that the Golgi structure in the empty vector groups and the S16A groups were damaged after OGD/R. Its morphology becomes more swollen, its spatial range is enlarged, and many small fragments form. However, the morphological changes of GA in S16D groups and WT groups were relatively less obvious, and the fragmentation of GA was reduced. These results suggest that phosphorylation of Hsp20 serine 16 site in mouse N2a cells and human sh-sy5y cells has a protective effect on Golgi fragmentation caused by OGD/R. Golgi stress is activated.

One of the key mechanisms of GA fragmentation is oxidative stress. Oxidative stress mediates GA fragmentation mainly through the following three pathways [10]: first, oxidative stress results in Golgi microtubule injury. Changes in the expression of GMAP210, which is a Golgi microtubule, were detected next. Second, oxidative stress activates apoptotic proteases, which remove Golgi structure proteins. Therefore, content changes in the Golgi structure proteins Golgin160 and Grasp65 were also detected. Third, oxidative stress induces calcium ion abnormalities, leading to GA fragmentation, which is caused by ER stress [11]. Therefore, changes in ER structure and function are detected last [10].

Hsp20 serine 16 site phosphorylation has a protective effect on GA morphology during OGD/R. Is this protective effect achieved by inhibiting Golgi stress response? To further clarify the protective mechanism of Hsp20 serine 16 site phosphorylation, Golgi stress related proteins were detected.

Grasp65 protein content was significantly lower than that of normal cells after OGD (P<0.05), but increased after OGD/R. Grasp65 is a Golgi structure protein that its phosphorylation can cause the fragmentation of GA by cyclin-dependent kinase-5 (CDK5) [12]. It is not that Grasp65 itself causes Golgi fragmentation. Therefore, we speculated that Grasp65 protein content declined because the cells could not withstand the blow of oxygen and sugar deprivation. However, as time went on, Golgi stress and ER stress played their roles, and phosphorylated Grasp65 protein content gradually increased. However, Grasp65 protein content in the WT groups were higher than that in the empty vector groups at 6 and 36 hours after OGD/R, indicating that the expression of Hsp20 could promote the increase of Grasp65 protein content after OGD/R.

GMAP210 protein content was significantly down-regulated compared with normal cells, either after OGR or after OGD/R. Studies have shown that GMAP210 is a Golgi microtubule-related protein with a molecular weight of 210kd, which is located in the cis-Golgi network [13]. The NH2 ends of GMAP210 are connected to the GA membrane, and the overexpression of cDNA encoding GMAP210 will lead to a sharp increase of GA’s morphology [14]. In contrast, the decrease of GMAP210 protein content leads to the fragmentation of GA [15]. In this study, decreased GMAP210 protein content was positively correlated with GA fragmentation. Moreover, after OGD/R, there was no significant difference in Golgi fragmentation among WT groups, S16D groups, empty vector groups and S16A groups. This result was consistent with increased expression of GMAP210 in WT groups and S16D groups after OGD/R. Therefore, our results confirmed that the protective effect of Hsp20 on GA in the OGD/R process was related to GMAP210.

The protein content of Golgin160 in the empty vector group was significantly lower than that in the normal cells group, and did not increase with the extension of reperfusion time. Golgin160 is a helical protein located on the cis-surface of the GA and can be cleaved by caspase during apoptosis. It is an important substance to maintain GA morphology and plays an important role in the process of cell apoptosis [16]. Therefore, the decreased protein expression of Golgin160 can lead to changes in GA morphology and Golgi fragmentation. After 24 and 36 hours of OGD/R, the content of Golgin160 protein in WT and S16D groups were significantly higher than that in the empty vector group and S16A groups. This means that Hsp20 can promote the increase of Golgin160 protein content during the OGD/R process, which can further promote the stabilization of GA morphology and reduce the occurrence of Golgi fragmentation.

These results indicated that the protective effect of Hsp20 serine 16 site phosphorylation on GA during OGD/R was completed by reducing the content of GA related proteins and inhibiting Golgi stress response.

In this study, after four plasmids were transfected into human sh-sy5y cells, the ER structure was still a closed network connected by the endometrium. However, the structure of the ER changed significantly after OGD/R. The ER structure in human sh-sy5y cells in the empty vector groups and the S16A groups were damaged after 24 h of OGD/R. The layered structure of the ER increases and the network structure decreases. However, these changes were not obvious in the WT groups and the S16D groups. These results suggest that Hsp20 can reduce the morphological changes of the ER caused by OGD/R. The protective effect of Hsp20 is due to phosphorylation at Hsp20 serine 16 site. The next question is how this happened.

In this study, Grp78 was significantly increased after OGD/R, suggesting that ER stress occurs in response to apoptosis after OGD/R. However, after reperfusion for 6 hours, 24 hours and 36 hours, Grp78 protein content in the empty vector groups continued to increase, while the protein content in the WT groups were relatively reduced. This suggested that ER stress was down-regulated and inhibited in the WT groups, and Hsp20 had a protective effect in the process. In the empty vector groups, ER stress continued to occur, eventually inducing apoptosis. The protein content of Grp78 in S16D groups were lower than that in S16A groups, suggesting that the cause of down-regulation of Grp78 protein content was phosphorylation of Hsp20. In general, OGD/R can induce ER stress. But Hsp20 can alleviate ER stress induced by OGD/R, and its protective effect is due to the phosphorylation on serine 16 site. Now it is clear that ER stress is activated, we will discuss how it is activated next.

Protein content of ATF6 and CHOP were significantly increased after OGD. These results indicated that after the isolation of ATF6 from GRP78, the ATF6 unfolded protein reaction signal was cascade initiated. The addition of ATF6 activated CHOP, and the production of CHOP increased significantly, eventually leading to apoptosis. The protein content of ATF6 and CHOP in the empty vector groups and S16A groups were significantly increased compared with that in the WT and S16D groups after 6, 24 and 36 hours of OGD/R. The protective effect of Hsp20 on nerve cells is mediated by ATF6 signaling pathway, and its protective effect is due to the phosphorylation of serine 16 site.

Compared with the normal group, phos-eif2 alpha 1 protein contents are significantly increased after 4 hours of OGD and 36 hours of reperfusion. The protein content of phos-eif2 alpha 1 protein in the WT groups and the S16D groups decreased by more than 50% compared with the empty vector group and the S16A groups after 36 hours of OGD/R. The protective effect of Hsp20 on nerve cells is also mediated by the PERK pathway, which is due to the phosphorylation of the serine 16 site. In summary, the phosphorylation of Hsp20 at the serine 16 site led to a decrease in the protein content of Grp78 after OGD/R, which further led to the reduction of ATF6 and phos-eif2 alpha 1. The down-regulation of ATF6 protein content further promoted the down-regulation of CHOP, which eventually reduced the cell death rate and made the ER structure more stable. After being separated from Grp78, ATF6 was transferred to GA, and Golgi proteasome could remove its transmembrane fragments.

Therefore, the ER may transmit signal through the ATF6 pathway, and induce cell death through it, resulting in Golgi fragmentation and changes in related proteins. That is just our previous research content.

In conclusion, Hsp20 has a protective effect on sh-sy5y cells and N2a cells after OGD/R, and the reason is the phosphorylation of serine 16. It protects sh-sy5y cells by inhibiting Golgi stress during OGD/R. It also alleviates the changes of ER morphology after OGD/R by inhibiting ATF6 and PERK pathways in ER stress. Phosphorylated Hsp20 may be explored as a therapy for treating these diseases. These findings will provide options for treating diseases that have the same or a similar pathological basis of cerebral ischemia-reperfusion injury.

## Supporting information

S1 Fig Raw data for Fig 7. (JPG)

S1 File Raw data for Figs 1–8. (XLSX)

## Abbrevations

GFP: green fluorescent protein
OGD/R: oxygen-glucose deprivation followed by reperfusion
sHsp: small heat shock protein
GA: Golgi apparatus
ER: Endoplasmic reticulum
GMAP210: Golgi microtubule-associated protein 210
PERK: PKR-like ER kinase
IRE-1: Inositol-requiring enzyme 1
ATF6: activating transcription factor 6
eIF2α: Eukaryotic translation initiation factor 2A
XBP1: X-box binding protein 1
Grp78: Glucose-regulated protein 78
CHOP: C/EBP-homologous protein
UPR: unfolded protein response
DAPI: 2-(4-Amidinophenyl)-6-indolecarbamidine dihydrochloride
