# Supplementary figures and images for: The mechanism on phosphorylation of Hsp20Ser16 inhibit GA stress and ER stress during OGD/R
Source: PLoS One. 2019 Mar 7;14(3):e0213410. doi: 10.1371/journal.pone.0213410 (PMC6405072; doi:10.1371/journal.pone.0213410)

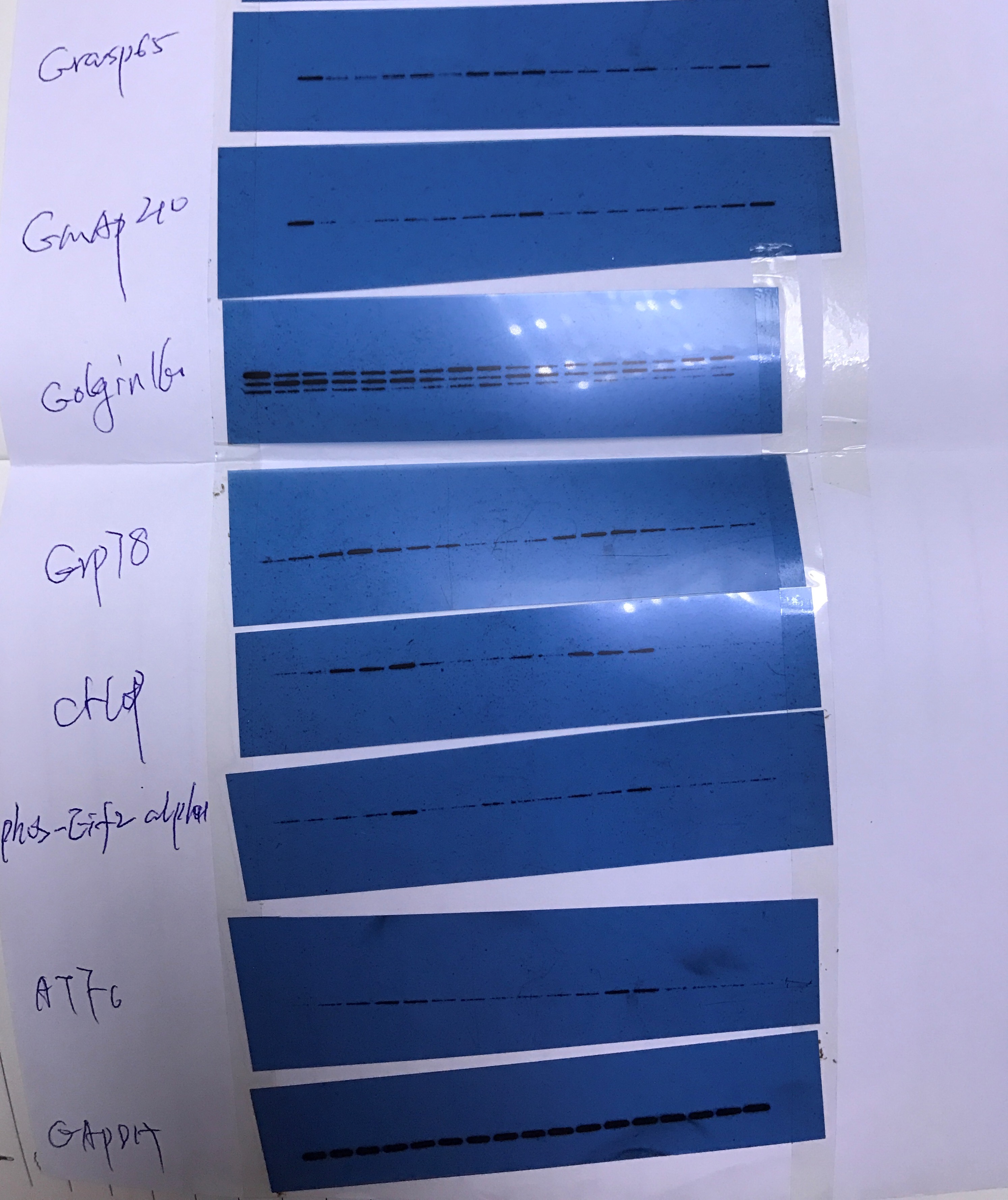

Supplement: S1 Fig — (JPG) [file pone.0213410.s001.jpg]
